# Incorporation of Collagen in Calcium Phosphate Cements for Controlling Osseointegration

**DOI:** 10.3390/ma10080910

**Published:** 2017-08-06

**Authors:** Ming-Hsien Hu, Pei-Yuan Lee, Wen-Cheng Chen, Jin-Jia Hu

**Affiliations:** 1 Department of Biomedical Engineering, National Cheng Kung University, Tainan 701, Taiwan; minghsienhu@gmail.com (M.-H.H.); b1208b@ms26.hinet.net (P.-Y.L.); 2 Department of Orthopedics, Show-Chwan Memorial Hospital, Changhua 50544, Taiwan; 3 Department of Orthopedic Surgery, Faculty of Medicine, National Yang-Ming University, Taipei 112, Taiwan; 4 Department of Fiber and Composite Materials, College of Engineering, Feng Chia University, Taichung 40724, Taiwan; 5 Medical Device Innovation Center, National Cheng Kung University, Tainan 701, Taiwan

**Keywords:** posterolateral lumbar fusion, non-dispersive calcium phosphate bone cement, collagen, osteogenesis

## Abstract

In this study, we investigated the effect of supplementing a non-dispersive dicalcium phosphate-rich calcium phosphate bone cement (DCP-rich CPC) with type I collagen on in vitro cellular activities and its performance as a bone graft material. Varying amounts of type I collagen were added during the preparation of the DCP-rich CPC. In vitro cell adhesion, morphology, viability, and alkaline phosphatase (ALP) activity were evaluated using progenitor bone cells. Bone graft performance was evaluated via a rat posterolateral lumbar fusion model and osteointegration of the implant. New bone formations in the restorative sites were assessed by micro-computed tomography (micro-CT) and histological analysis. We found that the incorporation of collagen into the DCP-rich CPC was associated with increased cell adhesion, cell viability, and ALP activity in vitro. The spinal fusion model revealed a significant increase in bone regeneration. Additionally, better osseointegration was observed between the host bone and graft with the DCP-rich CPC supplemented with collagen than with the collagen-free DCP-rich CPC control graft. Furthermore, compared to the control graft, the results of micro-CT showed that a smaller amount of residual material was observed with the collagen-containing DCP-rich CPC graft compared with the control graft, which suggests the collagen supplement enhanced new bone formation. Of the different mixtures evaluated in this study (0.8 g DCP-rich CPC supplemented with 0.1, 0.2, and 0.4 mL type I collagen, respectively), DCP-rich CPC supplemented with 0.4 mL collagen led to the highest level of osteogenesis. Our results suggest that the DCP-rich CPC supplemented with collagen has potential to be used as an effective bone graft material in spinal surgery.

## 1. Introduction

Over one million patients with skeletal issues require orthopedic surgery each year. Bone grafts are widely utilized in orthopedic surgery to augment bone union and regeneration [1]. The use of calcium phosphate cements (CPCs) as a synthetic bone material has been studied since the early 1980s [2]. CPCs are suitable bone-graft material because of their similarity in composition to the mineral phase of bone, as well as their high biocompatibility and osteoconductivity [3]. In our previous study, we evaluated the effectiveness of three calcium phosphate bone-graft substitutes in a rat model of posterolateral lumbar fusion and found that a dicalcium phosphate (DCP)-rich CPC, which has a Ca/P ratio as low as 1.5, had the best spinal fusion effectiveness [4].

Various strategies have been developed and evaluated for promoting the conversion of CPC to new bone, including increasing its porosity and using water-soluble polymers and platelet-rich plasma (PRP) as additives in apatite-forming CPCs [5,6,7]. A previous study showed that the incorporation of mineralized collagen into bone cement improved osteogenic ability, marked by increased differentiation of human marrow mesenchymal stem cells (MSCs) into osteoclasts and then osteoblasts [8]. Moreover, the addition of collagen to apatitic CPCs allows enhancement of several functional properties, such as significantly enhanced alkaline phosphatase (ALP) activity with collagen-containing nanosized hydroxyapatite [9]. In a study by R.A. Perez et al., collagen incorporation in the liquid phase of the CPC resulted in improved osteoinductive ability [10]. In addition, the adhesion and proliferation of osteoblastic cells increased in the calcium-deficient hydroxyapatite-collagen composite. These findings may be attributed to the reduced stiffness of the CPC as compared to collagen-CPCs composite in solid form. Great care must be taken when collagen-CPC grafts are applied during orthopedic surgery because the non-strengthened composite can easily be washed out by blood. We hypothesize that the incorporation of collagen into dicalcium phosphate (DCP)-rich CPC, a non-dispersive cement [11], may have several advantages, such as increased cell adhesion, proliferation, ALP activity, resistance to wash-out, and importantly, increased ability of MSCs to undergo osteogenic differentiation. 

The aim of this study was to investigate the effect of supplementing DCP-rich CPC with type I collagen on cellular activities and its performance as a bone graft material. Collagen solution and the DCP-rich CPC were mixed to create a moldable bone graft, which was then evaluated for cell adhesion, morphology, viability, and ALP activity using bone cell progenitors. The use of the bone graft in posterolateral lumbar fusion surgery in a rat model was assessed with radiology using three-dimensional micro-computed tomography (micro-CT). Finally, the optimal ratio of collagen to DCP-rich CPC was determined.

## 2. Materials and Methods

### 2.1. Preparation of the DCP-Rich CPC 

Tetracalcium phosphate (TTCP; Ca_4_(PO_4_)_2_O) powder was prepared via the sintering of dicalcium pyrophosphate (Ca_2_P_2_O_7_; Alfa Aesar, MA, USA) and calcium carbonate (CaCO_3_; Shimakyu’s Pure Chemicals, Osaka, Japan). The DCP-rich CPC was prepared by mixing TTCP and dicalcium phosphate anhydrous (DCPA; CaHPO_4_; Acros Organics, Geel, Belgium) powders at a DCPA-to-TTCP molar ratio of two. The detailed procedures for preparing DCP-rich CPC have been described previously [12].

### 2.2. Solubilized Type I Collagen

Concentrated type I collagen, derived from rat tail tendon (9.9 mg/mL), was purchased from BD Biosciences (Franklin Lakes, NJ, USA). Type I collagen was used because it is the main component of the organic part of bone.

### 2.3. Collagen-Containing DCP-Rich CPC

Collagen-containing DCP-rich CPC was prepared by mixing 0.8 g DCP-rich CPC powder (P) with 0.1, 0.2, and 0.4 mL type I collagen (S) solution to create mixtures with S/P ratios of 0.125, 0.25, and 0.5 mL/g, respectively. DCP-rich CPC with no collagen served as the control. The formation rate of collagen-containing DCP-rich CPC is not affected with the addition of collagen solution [4]. The samples were loaded into a 1 mL syringe with the needle removed for injection as per previous experiments on D1 cell attachment and L4–L5 posterolateral fusion in lumbar vertebrae models [12].

### 2.4. In Vitro Study

The D1 cells (bone-marrow derived MSCs from Balb/C mice) were purchased from the American Type Culture Collection. The cells were cultured in Dulbecco’s modified Eagle’s medium supplemented with 10% fetal bovine serum at 37 °C under a humidified 5% CO_2_ atmosphere, and were used before their eighth passage. The expression of ALP is a widely known osteogenic biomarker and has been commonly used in measuring the differentiation ability of progenitor bone cells. Cell proliferation and ALP activity were examined at one, four, and seven days after initial seeding of 1 × 10^5^ D1 cells on the surface of the specimen. The Alamar Blue VR assay (Bio-Rad Laboratories, Hercules, CA, USA) and *p*-nitrophenyl phosphate assay (Sigma-Aldrich, Saint Louis, MO, USA) were used to determine cell viability and production of ALP, respectively. These assays were performed according to instructions from the manufacturer. After the set culturing time, blank references for the Alamar Blue VR assay were measured to coincide with a cell viability of 0%. Cell optical density was read at a wavelength of 575 and 595 nm using a microplate enzyme-linked immunosorbent assay (ELISA) reader system (Spec384; Molecular Device, Sunnyvale, CA, USA). The ALP optical density at 405 nm was also determined. Each experiment was performed in triplicate (*n* = 3). Production of ALP was further verified by ALP staining with serum tartrate-resistant acid phosphatase and ALP double-stain kit (Takara Bio, Shiga, Japan). ALP-stained samples were washed prior to gross examination using light microscopy.

### 2.5. Morphology of Cell Attachment

D1 cells (1 × 10^5^ cells) were seeded onto the surface of the control sample and the DCP-rich CPC supplemented with type I collagen samples in 48-well plates. Morphology of the D1 cells in the different groups was observed using a scanning electron microscope (SEM; Hitachi S-3000N; Hitachi High-Technologies, Tokyo, Japan) equipped with energy dispersive spectroscopy (Horiba EX220; Horiba, Kyoto, Japan). After one hour, one day, and two days, the D1 cells were washed with 1 × PBS to remove all residual solution. The cells were then fixed with glutaraldehyde and the samples were gold coated and analyzed using SEM. 

### 2.6. In Vivo Study

#### 2.6.1. L4–L5 Lumbar Vertebrae Posterolateral Fusion Surgical Procedure

Forty Sprague Dawley (SD) rats (male, eight weeks old, 300–330 g) were used in this study. The rats were randomly divided into the control group and the group of 0.8 g DCP-rich CPC powder with 0.4 mL type I collagen according to the bone graft substitute to be implanted. The L4–L5 posterolateral fusion surgical procedure was performed after the rats were placed under general anesthesia by administration of tiletamine/zolazepam (Zoletil, 40 mg/kg bodyweight) and xylazine (5 mg/kg bodyweight) via intraperitoneal injection. The posterolateral lumbar fusion was performed following well-established procedures [13]. Briefly, the rats were placed in the prone position, and the surgical site was shaved and disinfected. A posterior midline incision of approximately 30 mm was made on the skin over the lumbar spine in the L4–L5 area. Subsequently, two linear incisions were made 3–5 mm laterally from the midline on the lumbar fascia. The soft tissue was carefully dissected, and the transverse processes of the L4 and L5 vertebrae were exposed. Bilateral transverse process decortication was performed using a low-speed burr until bleeding from the bone marrow could be verified. A block of properly-shaped DCP-rich CPC (0.1 mL) with or without type I collagen was placed between the transverse processes of the L4 and L5 vertebrae on each side. Finally, the fascia and skin incisions were closed with a 3-0 nylon suture. Postoperative pain was treated with ketoprofen (3 mg/kg body weight). The animals were monitored daily until they were sacrificed one to four weeks after surgery via a cardiac injection of ethanol. All procedures in this animal study were approved by the Institutional Animal Care and Use Committee of Show Chwan Memorial Hospital (Approval No. 102031). The National Institutes of Health guidelines for the care and use of laboratory animals were observed. Furthermore, we calculated the residual graft ratio between the L4–L5 transverse processes in order to evaluate CPC resorption in both groups. 

#### 2.6.2. Radiographic Analysis

Posteroanterior radiographs (In-Vivo Xtreme; Bruker Corp., Billerica, MA, USA) of the spine were taken one to four weeks post-operation to assess spinal fusion. Bone fusion between the transverse processes of the L4 and L5 vertebrae was independently evaluated by two orthopedic surgeons. The rats were sacrificed after radiographic examination. The lower part of the spine (L1–L6) was harvested for the following analyses. 

#### 2.6.3. Microcomputed Tomography

Micro-CT (SkyScan 1076, Bruker, Billerica, MA, USA) was performed on all specimens harvested. The residual ratio of graft-to-defect was calculated using image analysis software (Mimics; Materialise, Leuven, Belgium). The volume of residual implant on each side was calculated postoperatively. Residual graft ratio was calculated using the following equation: Residual graft ratio, % = [total volume of residual implant/total volume of original implant (0.1 mL)] × 100%.

#### 2.6.4. Histological Analysis

Specimens were fixed for 24 h in 10% formalin for histological analysis. The tissue was dehydrated with 50, 70, and 95% ethanol for one hour each, and with 100% ethanol for two hours. After dehydration, the tissue was left overnight at room temperature in the resin mixture, consisting of a 3:1 solution of methyl methacrylate and dibutyl phthalate. The tissue was then embedded in a polyethylene molding cup. Coronal sections were obtained from both sides of each rat (*n* = 3). Four-micron sections were cut and stained with hematoxylin and eosin (H&E) for analysis of general morphology and identification of the slides. Images were acquired with a light microscope (Leica DMI 3000B; Leica Biosystems, Wetzlar, Germany) equipped with a digital camera (Leica DFC295; Leica Microsystems, Wetzlar, Germany). Histological analysis was performed every week after surgery for both groups.

#### 2.6.5. Statistical Analysis 

One-way analysis of variance (ANOVA) in conjunction with Holm-Sidak post hoc testing was used to compare the results. In all cases, results were considered significantly different when *p* < 0.05.

## 3. Results

### 3.1. Cell Viability after Varying Culture Durations

**Figure 1:**
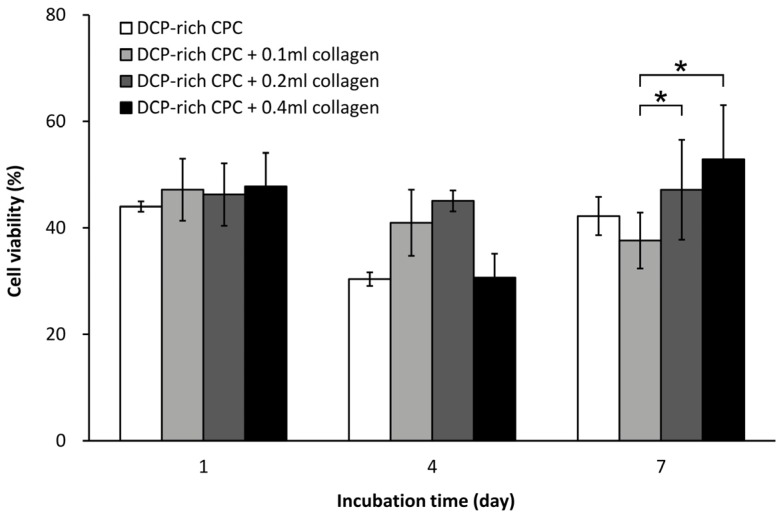
Cell viability of D1 cells seeded on different DCP-rich CPC samples after days 1, 4, and 7 of incubation (*n* = 3). * *p* < 0.05.

In this study, D1 cell viability was measured after incorporating varying amounts of collagen solution into the DCP-rich CPC. The DCP-rich CPC was found to be difficult in forming bone cement when the volume of the collagen solution volume exceeded 0.4 mL. Figure 1 shows cell viability of the different specimens. On day 1 of cell culture, there was no significant difference in cell viability among the specimens. Cell viability of the control and of the DCP-rich CPC supplemented with 0.1, 0.2, and 0.4 mL collagen solution was 30.37, 40.94, 45.06, and 30.65% at day 4 of culture, respectively, and 42.22, 37.63, 47.15, and 52.92% at day 7 of culture, respectively. Cell viability on the DCP-rich CPC supplemented with 0.2 mL collagen remained nearly unchanged from days 1 to 7. In the group where the DCP-rich CPC was supplemented with 0.1 mL collagen, cell viability progressively decreased from days 1 to 7. Although cell viability on the DCP-rich CPC supplemented with 0.4 mL collagen decreased at day 4, it was the highest at day 7 and was also significantly higher than those of the other groups. 

### 3.2. In Vitro Study

#### 3.2.1. Collagen Promotes Bone Marrow Cell Attachment to DCP-Rich CPC

**Figure 2:**
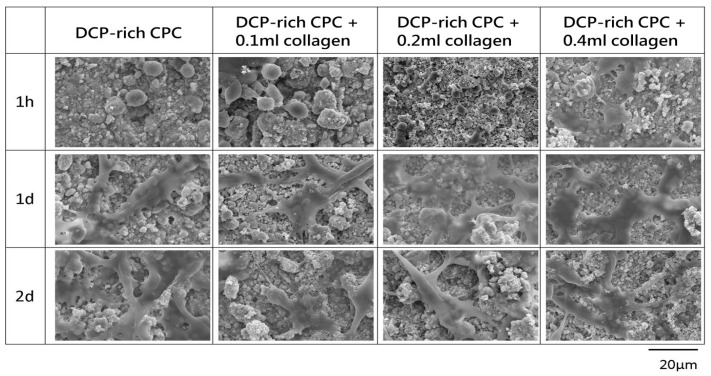
Typical morphologies of different DCP-rich CPC samples with fixed D1 cells, as evaluated by SEM.

Figure 2 shows the morphologies of D1 cells cultured in different DCP-rich CPC samples after one hour, one day, and two days. After one hour, the D1 cells on all samples extended their filopodia, which are thin and actin-rich cytoplasmic projections that act as the antennae of cells [14]. Notably, D1 cells on DCP-rich CPC with 0.4 mL collagen had greater extension of filopodia than D1 cells on the other samples at one hour. After one and two days of culture, cells with long extensions and overlapping cells were observed in all samples. The extensions are regions of cytoplasmic membranes, which contain a mesh-work or bundles of actin-containing microfilaments for cell-migration along a substratum [15]. The cells with filopodia were observed on the DCP-rich CPC supplemented with 0.4 mL collagen for one to two days longer than on the other samples. 

#### 3.2.2. ALP Measurements by the Respective Values of Cell Viability

**Figure 3:**
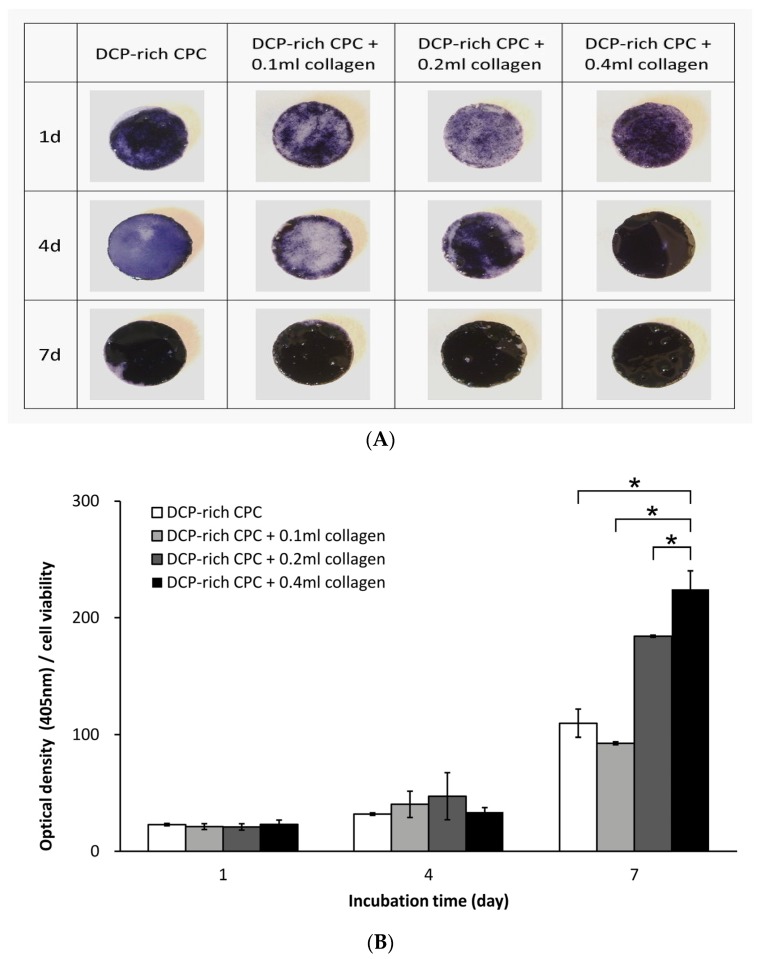
(**A**) ALP staining of D1 cells seeded on different DCP-rich CPC samples after days 1, 4, and 7 of incubation; (**B**) OD and ALP activity of each D1 cell seeded on different CPC samples after days 1, 4, and 7 of incubation (*n* = 5). * *p* < 0.05.

As shown in Figure 3A, the DCP-rich CPC group had the darkest staining at day 1. However, from day 4 to day 7, DCP-rich CPC with collagen samples showed darker blue staining than the control sample. The above mentioned results could be further verified by ALP semi-quantitative comparison per cell. Figure 3B shows the ALP absorbance values and ALP activity per cell were higher with the DCP-rich CPC supplemented with collagen than with the DCP-rich CPC alone for the different culture durations. Notably, ALP activity of the DCP-rich CPC supplemented with 0.1 mL collagen was not significantly different from that of the control at all measured time points, suggesting insufficient dosage of type I collagen. ALP activity of the DCP-rich CPC supplemented with 0.4 mL collagen was significantly increased from 23% on day 1 to 224% on day 7, and the measured activity was significantly different from that of the control on day 7. Early expression of ALP activity on the DCP-rich CPC supplemented with 0.4 mL collagen was not significantly different from the control on days 1 and 2.

### 3.3. In Vivo Study

#### 3.3.1. Radiographic Evaluation 

**Figure 4:**
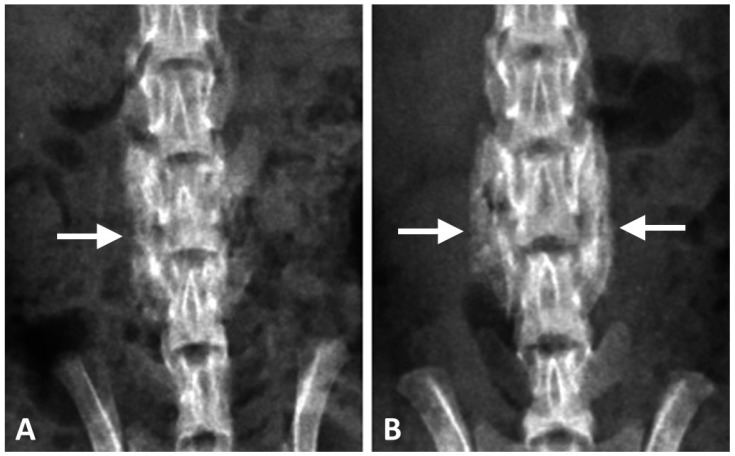
Posteroanterior radiographs after post-operative four weeks of the spines that underwent posterolateral lumbar fusion with (**A**) DCP-rich CPC or (**B**) DCP-rich CPC + 0.4 mL collagen. The arrows indicate fusion mass.

The results of the in vitro study showed that the DCP-rich CPC supplemented with 0.4 mL collagen led to the highest levels of cell viability, cell attachment, and ALP staining. Therefore, the DCP-rich CPC supplemented with 0.4 mL collagen was selected for comparison with control for testing in vivo. The two materials were used in L4–L5 posterolateral fusion in SD rats. Radiographs four weeks post-operatively revealed notable fusion mass in both groups. For the DCP-rich CPC supplemented with 0.4 mL collagen group, more fusion mass was clearly observed bilaterally in the L4–L5 interspace than in the control group (Figure 4). 

#### 3.3.2. Reduced Effect of Residual Graft Ratio on DCP-Rich CPC by Integrating Collagen

**Figure 5:**
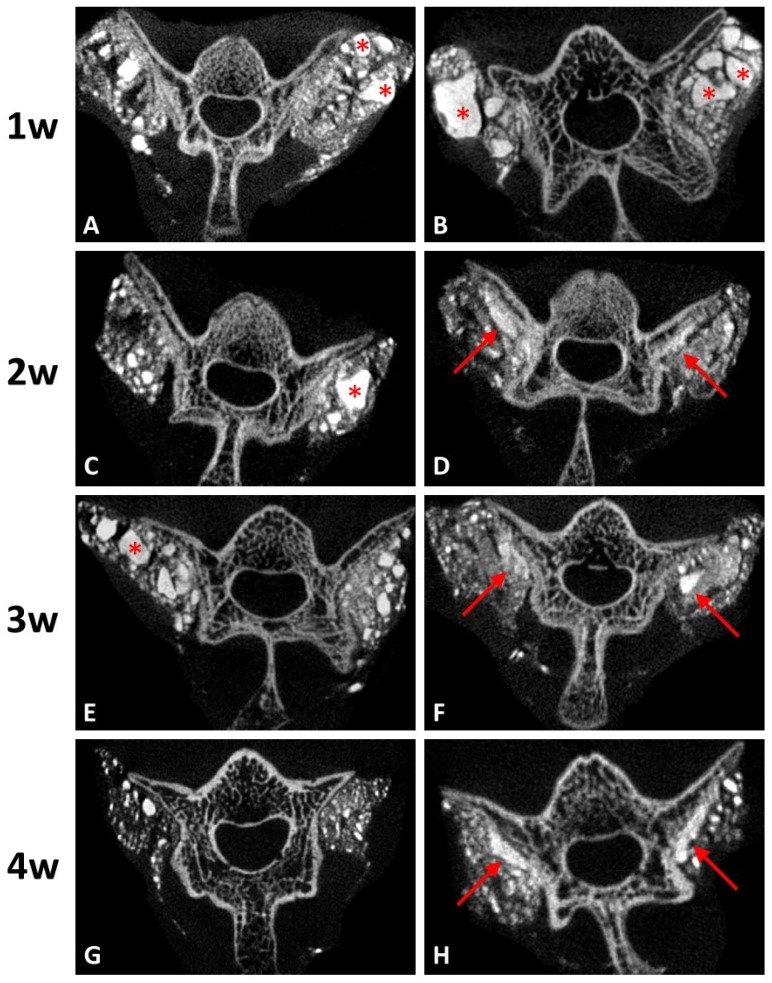
Coronal micro-CT images of the lumbar spines. (**A**,**C**,**E**,**G**) represent post-operative 1–4 weeks of DCP-rich CPC only, respectively. (**B**,**D**,**F**,**H**) represented post-operative 1–4 weeks of the DCP-rich CPC supplemented with 0.4 mL collagen, respectively. The star symbols indicate residual grafts and the arrows indicate callus formation.

Coronal micro-CT images at every stage in both groups are presented in Figure 5. At the first post-operative week, both groups had irregular shape of radiopaque lobules, which indicated the residual grafts (Figure 5A,B). After two weeks, micro-CT imaging of the group with DCP-rich CPC supplemented with 0.4 mL collagen showed that the radiopaque lobules became smaller and closer to the transverse process and lamina (Figure 5D,F). At the fourth post-operative week, the band-like callus formation with similar radiodensity to nearby spine structure was noted along the vertebra surface (Figure 5H). However, the same results were not observed in the group with DCP-rich CPC only. The radiopaque lobules remained scattered irregularly until the third week (Figure 5C,E). Although the residual grafts were absorbed slowly after four weeks, no band-like callus formation was noted (Figure 5G). 

**Figure 6:**
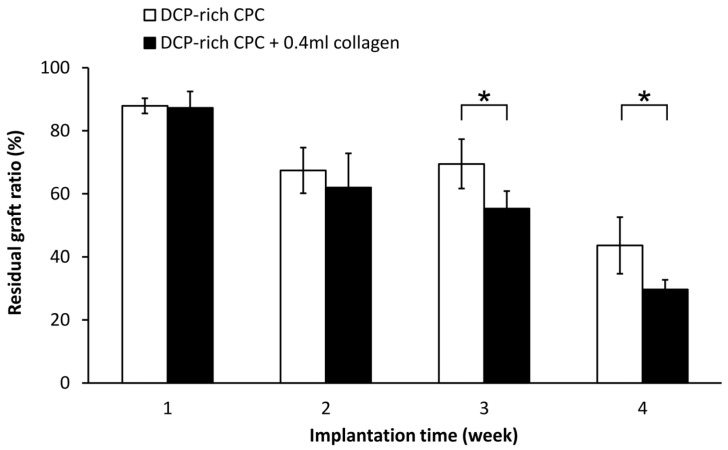
The comparison of residual graft ratio among two groups (*n* = 3). * *p* < 0.01.

Residual graft volume of both sides was measured through the contrast of the image in Figure 5. The residual graft ratio was significantly different between the control group and the DCP-rich CPC supplemented with 0.4 mL collagen group at three and four weeks after implantation (Figure 6). Notably, the residual graft ratio of the DCP-rich CPC supplemented with 0.4 mL collagen group was reduced to 55% at three weeks and 30% at four weeks. Meanwhile in the control group, the residual graft ratio was 70% at three weeks and 50% at four weeks. The residual graft ratio in the DCP-rich CPC supplemented with 0.4 mL collagen group was significantly lower than that in the collagen-free DCP-rich CPC group at both three and four weeks postoperatively (*p* < 0.01). 

### 3.4. Histology

**Figure 7:**
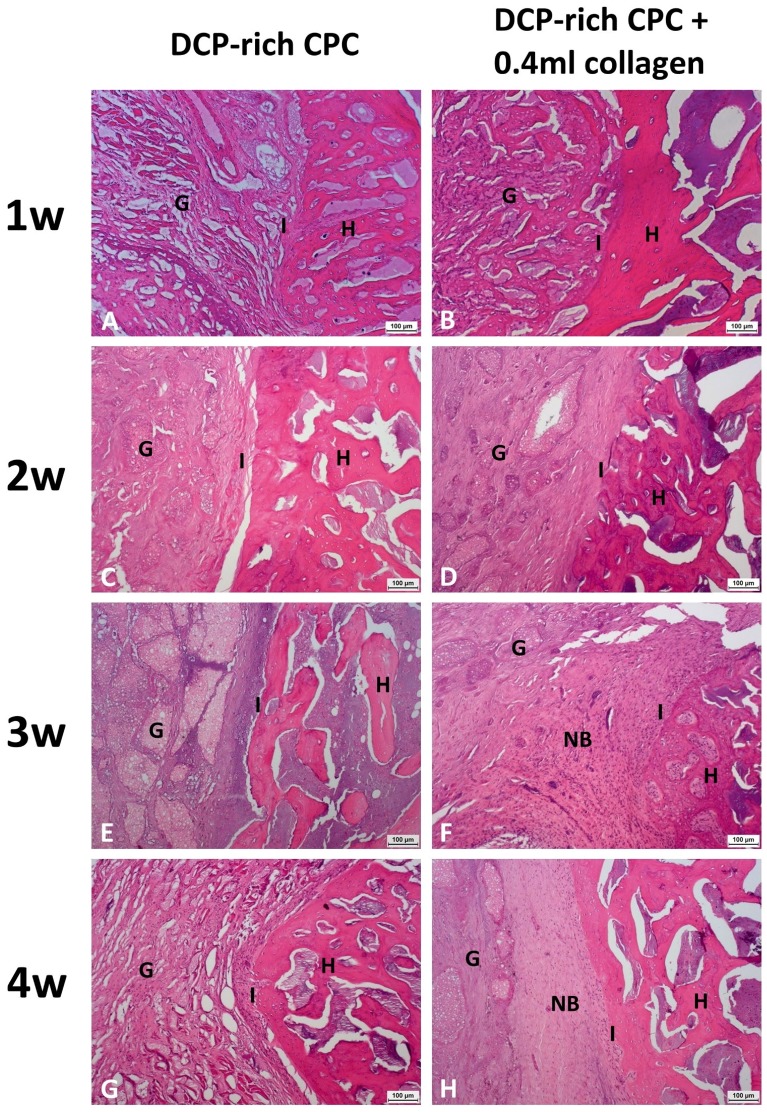
Representative histological coronal sections between the L4 and L5 transverse processes stained with haemotoxylin and eosin. (**A**,**C**,**E**,**G**) represented 1–4 weeks post-surgery of DCP-rich CPC group (100×). (**B**,**D**,**F**,**H**) represented 1–4 weeks post-surgery of DCP-rich CPC supplemented with 0.4 mL collagen group (100×). Scale bar = 100 μm. Abbreviation: G, implanted graft; H, host bone; I, interface between host bone and implanted graft; NB, new bone formation.

**Figure 8:**
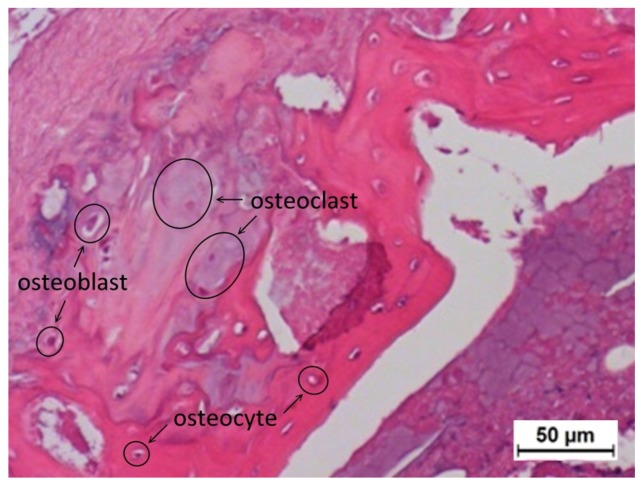
Representative histological image between the L4 and L5 transverse processes stained with haemotoxylin and eosin at four weeks post-surgery of the DCP-rich CPC supplemented with 0.4 mL collagen (200×). Osteoblasts are single-nucleus cells, found on the surface of bones, and separated from each other by their underlying matrix. Osteoclasts are characterized as large cells with multiple nuclei and ‘foamy’ cytosols. Scale bar = 50 μm.

We noted that the control DCP-rich CPC grafts readily dropped off from the implantation site during specimen preparing procedures, including during the dehydration and cutting steps (Figure 7, left panel). The observed cavities indicate that the control DCP-rich CPC graft did not transform into new bone to an appreciable extent, and therefore, was not well-fused to the vertebrae body. The lack of adhesive force resulted in the grafts being easily washed out or dropped off. Conversely, more tissue bridging was present at the interface between new bone formation and bone graft in the DCP-rich CPC supplemented with 0.4 mL collagen group one to four weeks postoperatively than in the control group (Figure 7). Specifically, at postoperative week 1 there was no significant difference observed between the two groups. At week 2, the graft-bone junction was wider in the control group than in the DCP-rich CPC supplemented with collagen group with no other observed differences between groups. At weeks 3 and 4, new bone formation as well as activated osteoblasts and osteoclasts were noted in the DCP-rich CPC supplemented with 0.4 mL collagen group, which was not observed in the control group (Figure 8). 

## 4. Discussion

Autologous bone grafting has been the gold standard treatment in posterolateral lumbar fusion for many decades; however, issues associated with the use of autografts include limited availability and variability of graft quality, increased risk of hematoma, infection, and bleeding, increased operative time, chronic donor site pain, and high cost [16]. Subsequently, much effort has been devoted to the development of novel bone graft substitutes [17]. Similar composition to the mineral phase of bone as well as good biodegradability, bioactivity, and osteoconductivity support the rationale behind the use of calcium phosphate as a bone substitute. Among the different CPCs, DCP-rich CPC with a Ca/P ratio of 1.50 shows great potential for success in spinal fusion [4]. While a previous study showed an increase in osteogenic ability of bone repair with incorporation of collagen into bone cement [6], the optimal amount of collagen to maximize cell viability has not yet been determined, especially for DCP-rich CPC. The incorporation of solubilized collagen increases the collagen concentration of CPCs without reducing workability. CPC containing 1% solubilized collagen has been shown to have the highest adhesion while promoting proliferation and differentiation of human osteoblast-like SAOS-2 cells [10]. Study results support that the effect on mechanical properties of CPC from the addition of collagen is dependent on collagen concentration. 

Previous studies have revealed that mouse MSCs show higher ALP activity and higher mRNA expression of type I collagen during osteogenic differentiation, and that type I collagen can induce MSC differentiation in vitro [18,19,20]. Our study found that the addition of collagen promoted attachment of D1 cells onto the surface of DCP-rich CPC and increased cell viability. The D1 cells had greater attachment on the DCP-rich CPC with 0.4 mL collagen of bone graft substitute than on DCP-rich CPC only bone graft substitute on day 2; the phenomenon was not apparently observed with 0.1 or 0.2 mL collagen added. In a cell model, we observed that D1 cell viability initially decreased with the DCP-rich CPC supplemented with 0.4 mL collagen by day 4, followed by an increase from day 4 to day 7. Upon further observation, ALP activity and viability ratio of D1 cells significantly increased from day 4 to day 7. Our results show that DCP-rich CPC supplemented with collagen better elicited the early stage of progenitor D1 cell differentiation on surfaces than DCP-rich CPC without collagen. The results may be attributed to weaker initial adherence ability of D1 cells during the first four days. 

Our study demonstrated that the addition of 0.4 mL of 0.1% type I collagen to 0.8 g DCP-rich CPC enhanced osteointegration in the space between host bone and graft in a posterolateral lumbar fusion model. Histologically, a larger quantity of osteoblast-deposited lamellae of new bone tissue and a greater number of osteocytes were observed in the DCP-rich CPC supplemented with the collagen group than in the DCP-rich CPC group. It has been noted that the ions released from CPCs could cause ion–dipole interaction, cell adhesion, proliferation, and differentiation [12]. The biochemical signaling molecules secreted by osteocytes and osteoblasts to stimulate osteogenic differentiation of MSCs has also been well-recognized [21]. Therefore, the results might suggest that rapid CPC resorption could be achieved with a low Ca/P formula of DCP in CPC at an early stage. Our study found that regenerated bone volume at the implantation site was considerably greater in the DCP-rich CPC with 0.4 mL collagen group than in the control group, suggesting that the implanted DCP-rich CPC with 0.4 mL collagen largely transformed into bridging callus. Bone healing versus graft resorption was detectable and several calcium phosphate remnants were surrounded by provisional connective tissue, which would be eventually resorbed and transformed into new alveolar bone in situ.

There were several limitations in this study. Firstly, the in vitro study only analyzed three concentrations of collagen-containing DCP-rich CPC. The most effective concentration of DCP-rich CPC supplemented with collagen might not be included. However, we hope this study could be an anchor for following research. Secondly, only the DCP-rich CPC supplemented with 0.4 mL collagen was tested in the in vivo study because it resulted in the highest levels of cell viability, cell attachment, and ALP staining. However, if the in vivo study also included the group of the DCP-rich CPC supplemented with 0.1 mL or 0.2 mL collagen, we might obtain a better understanding of the effect of osteogenesis of type I collagen.

In this study, we systematically evaluated the effect of added collagen to the biological performance of the DCP-rich CPC and its ability to affect osteogenic differentiation in vitro and in vivo. Our results show that collagen not only promoted D1 cell attachment and proliferation, but that it also elicited the early stage of progenitor cell differentiation in terms of morphological characteristics and ALP activity. The DCP-rich CPC supplemented with collagen was also associated with a significantly increased bone turnover rate of L4–L5 transverse processes as shown via micro-CT. Overall, our results suggest that the incorporation of collagen promotes absorption of the DCP-rich CPC by osteoclasts as well as the laying down of a new osteoid matrix by osteoblasts, and that it has potential to be an effective treatment for use in bone defect restoration in spinal diseases.
